# Determinants of intimate partner violence during pregnancy among married women in Abay Chomen district, Western Ethiopia: a community based cross sectional study

**DOI:** 10.1186/s12905-016-0294-6

**Published:** 2016-03-10

**Authors:** Bedilu Abebe Abate, Bitiya Admassu Wossen, Tizta Tilahun Degfie

**Affiliations:** Department of Social and Public Health, College of Health Sciences, Debre Tabor University, Debre Tabor, Ethiopia; Department of Population and Family Health, College of Health Sciences, Jimma University, Jimma, Ethiopia; Institute of Development and Policy Research, Population and Gender Unit, Addis Ababa University, Addis Ababa, Ethiopia

**Keywords:** Intimate partner violence, Pregnancy, Ethiopia

## Abstract

**Background:**

Intimate partner violence during pregnancy is the most common form of violence that harms the health of women and the fetus but practiced commonly in developing countries. There is scarcity of information regarding intimate partner violence during pregnancy in Ethiopia. Thus, this study aimed to assess the prevalence and associated factors of intimate partner violence during recent pregnancy in Abay Chomen district, Western Ethiopia.

**Methods:**

Community based cross sectional study was conducted among married pregnant women in Abay Chomen district in April, 2014 using a standard WHO multi-country study questionnaire. Two hundred eighty two randomly selected pregnant women aged 15–49 years participated in the study. Logistic regression and multivariate analysis were employed.

**Results:**

The prevalence of intimate partner violence during recent pregnancy was 44.5 % (95 % CI, 32.6, 56.4). More than half 157 (55.5 %) experienced all three forms of intimate partner violence during recent pregnancy. The joint occurrence of intimate partner physical and psychological violence during recent pregnancy as well as joint occurrence of intimate partner physical and sexual violence was 160 (56.5 %). Pregnant women who were ever lived with their partner’s family were 46 % less likely to experience recent intimate partner violence. Dowry payment decreases intimate partner violence during recent pregnancy (AOR 0.09, 95 % CI 0.04, 0.2) and pregnant women who didn’t undergo marriage ceremony during their marriage were 79 % are less likely to experience violence (AOR 0.21, 95 % CI 0.1, 0.44).

**Conclusion:**

Nearly half of interviewed pregnant women experienced intimate partner violence during pregnancy implying the prevalence of such practice in the study site. To that end, increasing community awareness about the consequences of the practice could be important. Moreover, as health extension workers works closely with households, they could be crucial players to increase community awareness about intimate partner violence on pregnant mothers and halt it or its risk factors.

## Background

Declaration on the elimination of violence against women defines violence against women as “…any act of gender-based violence that results in, or is likely to result in, physical, sexual or psychological harm or suffering to women, including threats of such acts, coercion or arbitrary deprivation of liberty, whether occurring in public or in private life” [1]. By year 2013, one out of three women experienced violence [2]. Among those violence, intimate partner violence on pregnant women is a global health concern [2]. The WHO multi-country study on women’s health and domestic violence against women found the prevalence of physical intimate partner violence as 15 % in Japan to 71 % in Ethiopia and the prevalence of physical domestic violence against women in pregnancy 1 % in Japan city to 28 % in Peru Province, with the majority of sites ranging from 4 to 12 % [3]. Prevalence of violence against pregnant women in developing countries is estimated to be 4 to 29 % [4].

Analysis of demographic and health surveys and the international violence against women survey showed prevalence of intimate partner violence during ‘pregnancy to be 2 % in Australia, Denmark, Cambodia and Philippines to 13.5 % in Uganda, with the majority 4 and 9 % [5].

In Ethiopia, community based studies indicated that 50 to 76.5 % of women experienced domestic/intimate partner violence in their life time [6–10]. Prevalence of intimate partner physical violence during pregnancy in rural Ethiopia is reported to be 8 % [3].

Intimate partner violence during pregnancy has been associated with fatal and non-fatal adverse health outcomes for the pregnant woman and her unborn fetus either direct physical trauma to her body and physiological stress from current or past abuse or fetal growth and development [11–15].

Different studies showed that intimate partner violence against pregnant women was significantly associated with adverse maternal health outcomes i.e. unintended pregnancies, pregnancy-related symptom distress, inadequate prenatal care, induced abortion, spontaneous abortion, gestational weight gain, intra uterine restriction, hypertension, pre-eclampsia, third trimester bleeding and STIs. Pregnant women were at higher risk of adverse outcomes including maternal death [16–18].

In low and middle income countries including Ethiopia, there are several gaps and inadequate evidence on prevalence and factors associated with intimate partner violence during pregnancy [4]. Most studies were limited to intimate partner violence to non pregnant women. However, the consequence of intimate partner violence during pregnancy is worse.

Thus, this study assessed magnitude and associated factors of intimate partner violence during pregnancy (IPVDP) in Abay Chomen district, western Ethiopia.

## Methods

### Study area

The study was conducted in Abay Chomen district, Oromia region, western Ethiopia. Abay Chomen district is located in Horro Gudurru Wollega Zone of Oromia region, 246 Km to the west of Addis Ababa, the capital city of Ethiopia. The district has two administrative towns and 18 rural kebeles, which is the smallest administrative unit in Ethiopia. There were five health centers and 18 health posts in the study area [19].

### Study design and population

Community based cross sectional study was conducted in the district in April, 2014. Study population were randomly selected and pregnant women aged 15–49 years who are living in the study area for at least six months prior to the study were targeted.

Sample size was calculated using a single population proportion formula using the following assumptions; 8 % prevalence of intimate partner violence during pregnancy in rural Ethiopia (3) 95 % confidence level, 3 % margin of error, 10 % allowance for non-response rate. Accordingly, the total sample was 299.

Out of twenty one kebeles of Abay Chomen district, ten kebeles were randomly selected. Updated ANC registration of health extension workers was checked and household pregnant women census was done for one week by six interviewers and numbering was done in the selected kebeles to fix a sampling frame.

A total of 2021 pregnant women who lived at least six months prior to the study period were enumerated. Finally, population proportion allocation was done to identify respondents from the selected households as a study unit. In situations where households had two or more eligible subjects, only one was selected by lottery method.

### Data collection method and tools

Data was collected by six high school completed female interviewers using standard WHO multi-country study of VAW questionnaire [20]. The questionnaire was translated to local language Afan Oromo by experts in both languages and was translated back to English by another person to ensure consistency and accuracy. The data collection process was closely supervised by two health officers and the principal investigator. The data collectors and supervisors were recruited based on previous experience on data collection and fluency in the local language. In addition, training was given for two consecutive days on how to interview, handling ethical issues and maintaining confidentiality and privacy. Pre-test study covered 15 pregnant women in a separate kebele, which become out of the main study.

Pre-test was conducted to familiarize enumerators with the administration of interview process and for ensuring consistency. Debriefing sessions were held with the pre test field staff and the questionnaires were modified based on lessons drawn from the pre -test.

### Data analysis

Data was first checked manually for completeness and then coded and entered in to Epi data version 3.5.1. Then the data was exported to SPSS version 16.00 for data checking, cleaning and logistic regression. Cleaning was done by calculating frequencies and sorting. Bivariate analysis between dependent and independent variables was performed using binary logistic regression. *P* < 0.25 was used as criteria to select candidate variables for multivariate analysis. Multivariable logistic regression analysis was done to adjust for possible confounding variables. *P*-value < 0.05 with 95 % confidence interval (CI) for OR (odds ratio) was used in judging the significance of the associations. Results were presented in text, tables and charts.

### Ethical considerations

Ethical approval was obtained from Ethical Review committee of Jimma University. Letter of permission was obtained from Abay Chomen district administration and health offices. The purpose of the study was explained to the study participants and written consent was secured before data collection was started and confidentiality of the information was ensured by coding. For study participants aged less than 18, we received written informed consent from the participants themselves because they were married and mature minor. The consent procedure was approved by the ethics committee for all including those aged less than 18 years [21]. The interview with the pregnant women was undertaken privately in separate area within the household compound and fieldworkers were trained to refer women requesting assistance to available sources of support according to WHO Ethical and safety recommendation [22]. However, no pregnant women requested support.

## Result

### Socio demographic characteristics of pregnant women

A total of 282 out of 299 study subjects were interviewed making a response rate of 94.3 %. Majority 135 (47.7 %) of respondents were in the age range of 25–34 years. The mean age of the respondents’ was 27 years (±6.1SD). About two thirds of the respondents’ wealth quantile was medium. Nearly half 145 (51.8 %) of the respondents had no formal education. About two thirds 184 (65.0 %) were housewives, and half 143 (50.5 %) of them were grew up in the same community as they born or nearby community/Kebele (Table 1).

**Table 1:** Socio demographic characteristics of currently married pregnant women, Abay Chomen District, Western Ethiopia, April 2014 (*n* = 282)

Variable		Number (%)
Age (years)	15–24	112 (39.6)
	25–34	135 (47.7)
	35–44	36 (12.7)
Religion	Protestant	192 (67.8)
	Orthodox	82 (29.0)
	Others^a^	8 (2.9)
Ethnicity	Oromo	232 (82.0)
	Amhara	43 (15.2)
	Others^b^	7 (2.5)
Wealth Quintile	Poor	79 (28.0)
	Medium	185 (65.6)
	Rich	18 (6.4)
Growth areas (refers to where the mother grows before 12 years of age)	The same community	143 (50.5)
	Other Kebele	66 (23.3)
	Other town	40 (14.1)
	Others^c^	33 (11.6)
Occupation	House wife	184 (65.0)
	Daily laborer	39 (13.8)
	Private employee	19 (6.7)
	Gov employee	25 (8.8)
Education status	Illiterate	145 (51.6)
	Elementary	83 (29.5)
	High school	43 (15.3)
	Higher education graduate	10 (3.6)
Live with husband family	Yes	137 (48.4)
	No	145 (51.2)
Live with her family	Yes	233 (82.3)
	No	46 (16.3)

^a^Muslim, Wakefeta

^b^Tigre, Sidama, and Kembata

^c^Don’t remember/don’t know, other neighbor town, refused to answer

About half (52.3 %) reported that they had seen women being battered in their childhood and 32 (11.4 %) of them reported that they support pregnant women to be battered by her intimate partner. More than eight in ten 242 (85.5 %) had a person to whom they might talk when they encounter problem. Forty eight (17.0 %) were advised by the person to whom they talk to take violence as it is normal, where as 6 (2.1) and 4 (1.1 %) were advised to go to elders and to ask for divorce respectively.

Nearly nine out of ten 50 (88.3 %) of respondents marriage had dowry payment, which they consider has positive impact on how they are treated by their husband and his family and 226 (80.1 %) of them reported that the entire dowry was paid. Majority 205 (73.7 %) of them reported that dowry payment had positive impact on their rest of life. One in three 98 (34.6 %) of the conflict between the husband and the pregnant women was solved by the elders.

### Socio demographic characteristics of the current partner

Majority of respondents’ partners were between the age of 25–34 157 (55.5 %). About four in ten 106 (37.5 %) of partners had no formal education. Regarding occupation, 31.8 and 29.3 % of partners were government employees and farmers respectively. More than one in ten 37 (13.1 %) of them had other wife and 21 (7.4 %) had child from other wife. Nearly nine out of ten partners 251 (88.7 %) provided money for their wives. Nearly half 152 (48.1 %) of them drunk alcohol and one in three 88 (31.1 %) drunk 1–2 times a week and more (Table 2).

**Table 2:** Socio demographic characteristics of intimate Partner as reported by women, Abay Chomen District, Western Ethiopia, April 2014 (*n* = 282)

Variable		Number (%)
Age (years)	18–24	25 (8.8)
	25–34	157 (55.5)
	35–49	79 (27.9)
	>50	22 (7.8)
Education	Illiterate	106 (37.4)
	Elementary	68 (24.0)
	High school	71 (25.1)
	Higher education graduate	37 (13.1)
Occupation	Farmer	83 (29.3)
	Gov. employee	90 (31.8)
	Daily laborer	28 (9.9)
	Merchant	20 (7.1)
	Priv. employee	13 (4.6)
	Others^a^	12 (4.2)
Had other wife	Yes	37 (13.1)
	No	244 (86.2)
Alcohol frequency	Always	20 (7.1)
	1–2 times a week	68 (24.0)
	1–3 times a month	25 (8.8)
	Not drunk	147 (51.9)
Ever fight with others	Yes	55 (19.4)
	No	223 (78.8)
Had other child	Yes	21 (7.4)
	No	259 (91.5)

^a^Evangelist, Retired, Student

### Reproductive characteristics of pregnant women

One hundred forty two (50.2 %) of the respondents married in the age between 20–24 years with the mean age of first marriage 19.6 years (±2.9SD). Nineteen (42.4 %) of the couples the initiation of marriage was not based on their own choices and one in five 58 (20.5 %) were not volunteer to marry their current partner. Similarly, 95 (33.8 %) of them had never conducted marriage ceremony. Thirty seven (13.1 %) of them married to partners who had other wife. The mean age at first sex was 17.9 years (±2.35SD) (Table 3).

**Table 3:** Reproductive characteristics of currently married pregnant women aged 15–49, Abay Chomen District, western Ethiopia, April 2014 (*n* = 282)

Variable		Number (%)
Gravida	One	106 (37.5)
	Two	88 (31.1)
	> = Three	59 (31.5)
Marriage sequence	First	252 (89.4)
	Second	22 (7.8)
	Third and fourth	8 (2.9)
Marriage Ceremony	Religious	58 (20.6)
	Customary marriage	47 (16.7)
	Civil marriage	82 (29.1)
	No marriage ceremony	95 (33.7)
Who choose her husband	Both	150 (53.0)
	Husband	82 (29.0)
	His family	18 (6.4)
	My family	17 (6.0)
	I myself	13 (4.6)
Voluntariness to marry him	Yes	220 (77.7)
	No	58 (20.5)
Dowry /Bride payment	Yes	250 (88.3)
	No	5 (1.8)
	Don’t know	17 (6.0)
	Refused	10 (3.5)
Dowry/bride	All paid	226 (79.9)
	Partially paid	24 (8.5)
	Don’t know	21 (7.4)
	Refused	10 (3.5)
Dowry Impact	Positive impact	206 (73.8)
	Negative impact	3 (1.1)
	Nothing	45 (16.1)

NB: Dowry/bride: refers to dowry/bride price payment to the bride’s family in country with this culture like Ethiopia

Dowry impact: refers to on how the pregnant women are treated by their husband and his family

### Prevalence of violence and its forms

**Fig. 1:**
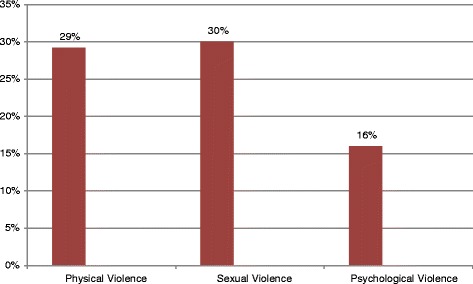
Forms of intimate partner violence during pregnancy, Abay Chomen district, Western Ethiopia, April 2014

Intimate partner violence during recent pregnancy was 44.5 %. More than half 157 (55.5 %) women experienced all the three forms of intimate partner violence during recent pregnancy. The simultaneous occurrence of intimate partner physical and psychological violence during pregnancy as well as joint occurrence of intimate partner physical and sexual violence was 160 (56.5 %) (Fig. 1).

### Physical violence

The prevalence of intimate partner physical violence during recent pregnancy (IPVDP) was 29.2 %. The commonly reported type of physical violence was batter 34 (41 %) followed by hit with fist/something else that could hurt them 18 (21.7) and 25 (8.8 %) had reported scar or wound (Table 4). The abdominal beat during recent pregnancy was 38 (13.4), and 72 (25.4 %) were beaten before pregnancy.

**Table 4:** Types of physical violence’s during pregnancy among currently married women aged 15–49, Abay Chomen District, Western Ethiopia, April 2014 (*n* = 282)

Type of physical violence	Count (%)
Battered	34 (41 %)
Pushed or shoved	6 (7.2 %)
Slapped or threw something at them	15 (18.1 %)
Hit with fist/something else that could hurt her	18 (21.7 %)
Kicked, drugged or beat her	5 (6.0 %)
Strangled, choked or burnt her on the purpose	3 (3.6 %)
Threatened to use knife or gun	2 (2.4 %)

### Sexual violence

The prevalence of intimate partner sexual violence during recent pregnancy was 85 (30.2 %). About 56 (19.9 %) of the respondents reported that their partners had forced them to have sexual intercourse without their interest or consent during recent pregnancy. In addition, 84 (29.9 %) of respondents experienced sexual intercourse during their pregnancy due to fear of their partners.

### Psychological (Emotional) violence

The prevalence of psychological/emotional intimate partner violence during recent pregnancy was 46 (16.3 %). Nearly four in ten 18 (37.5 %) of the participating women were verbally insulted and made feel bad about themselves at least once during recent pregnancy. The proportion of both pregnant women humiliated in front of other persons and insisted on them knowing where they were all times was 7 (2.5 %). The percentage of pregnant women who reported stress and depression as a result of violence were 24 (8.5) and 13 (4.6 %) respectively (Table 5).

**Table 5:** Different types of psychological violence’s among currently married women aged 15–49,, Abay Chomen District, Western Ethiopia, April 2014 (*n* = 282)

Type of Psychological violence	Number (%)
Insists on knowing where she go all the time	18 (37.5 %)
Belittled or humiliated in front of others	7 (14.6 %)
Tried to prevent her from seeing family or friends	7 (14.6 %)
Tried to prevent her from seeing other men	5 (10.4 %)
Scared or intimidated her on purpose	5 (10.4 %)
Blaming for all things	3 (6.2 %)
Suspicious for that she were unfaithful	1 (2.1 %)
Threatened to hurt her or someone she care about	2 (4.2 %)

### Factors associated with IPVDP

Result of binary logistic regression showed that childhood growth areas, partner’s education, partner’s age, age of marriage, age at first sex, ever lived with partner’s family, type of marriage ceremony, dowry payment, dowry impact in future life, having discussant to talk their problem, battering seen during childhood, support of pregnant women battering, money provision and marriage level were identified as significant predictors of experiences of IPVDP (intimate partner violence during recent pregnancy) while respondents’ educational status and occupation were not associated.

In multivariable logistic regression three variables i.e. dowry payment impact, partner education and undergoing marriage ceremony were associated. When compared with literates, illiterate partners were 50 % less likely to use violence against their intimate partner during recent pregnancy (AOR 0.5, 95 % CI 0.2, 0.9).

Pregnant women who reported that dowry payment has positive impact on how they are treated by their husband and his family were 8.7 times more likely to experience IPVDP than those who reported no positive impact on how they are treated by their husband and his family (AOR 8.7, 95 % CI 4.2, 17.9). Pregnant women who didn’t undergo marriage ceremony during their marriage were 4.1 times more likely to experience IPVDP (AOR 4.1, 95 % CI 2, 8.2) (Table 6).

**Table 6:** Factors associated with intimate partner violence during pregnancy among currently married women aged 15–49, Abay Chomen District, Western Ethiopia, April 2014 (*n* = 282)

Variable	Intimate partner violence during pregnancy				
	Yes	Yes (%)	No (%)	COR (95 % CI)	AOR (95 % CI)
Husband education	Illiterate	56 (52.8)	50 (47.2)	1.7 [1,2.8]	0.5 [0.2.0.9]
	Literate	69 (39.4)	106 (60.6)	1	1
Live with husband family	Yes	49 (35.8)	88 (64.2)	0.49 [0.3,0.8]	1.7 [0.9,3.1]
	No	76 (52.8)	68 (47.2)	1	1
Dowry impact	Positive	68 (33.2)	137 (66.8)	0.17 [0.09,0.3]	8.7 [4.2.17.9]
	Not positive	125 (44.3)	20 (26)	1	
To whom they talk violence	Yes	102 (42.3)	139 (57.7)	0.5 [0.2,1]	0 [0,∞]
	No	23 (59)	16 (41)	1	1
Batter seen during childhood	Yes	82 (55.4)	66 (44.6)	2.6 [1.5,4.2]	1.6 [0.93,3.1]
	No	43 (32.3)	90 (67.7)	1	1
Pregnant women supporting batter during pregnancy	Yes	21 (65.6)	11 (34.4)	2.6 [1.2,5.77]	0.4 [0.16.,1]
	No	103 (41.7)	144 (58.3)	1	1
Money provision of partner	Yes	115 (46)	135 (54)	0.53 [0.24,1.1]	1.9 [0.77,4.7]
	No	10 (32.3)	21 (67.7)	1	1
Marriage frequency	First	108 (43)	143 (57)	0.57 [0.26,1.2]	1.1 [0.45,2.9]
	Other	17 (56.7)	13 (43.3)	1	1
Marriage Ceremony	No	28 (29.5)	67 (70.5)	0.38 [0.22,0.64]	4.1 [2,8.2]
	Yes	97 (52.2)	89 (47.8)		0
Husband age	18–24	8 (32)	17 (68)	0.29 [0.08,0.97]	0.47 [0.11,1.9]
	25–34	69 (43.9)	88 (56.1)	0.48 [0.18,1.2]	0.46 [0.15,1.3]
	35–49	35 (44.3)	44 (55.7)	0.49 [0.18,1.3]	0.48 [0.15,1.5]
	> = 50	13 (61.9)	8 (38.1)	1	1

## Discussion

The study showed that intimate partner violence during recent pregnancy was 44.5 % which was consistent with study from Zimbabwe [23] and higher than studies conducted in USA [24], Japan, and South Africa. This could be because this study included psychological violence as an addition for measuring intimate partner violence during pregnancy [3, 25, 26] unlike the other studies. The higher prevalence found in this study also might indicate that women were disadvantaged segment of the population and the country’s patriarchal society norms.

About three in ten 82 (29.2 %) respondents experienced physical violence during recent pregnancy by their intimate partner. It is in agreement with study conducted in Namibia [3] and Tanzania. However, this finding is higher than finding from previous studies in Ethiopia and Serbia and Montenegro [3]. The possible reason might be the presence of traditional norms that support beating pregnant women in the study area because 32 (11.4 %) of the respondents reported that they support pregnant women to be battered by her intimate partner.

Likewise, in this study 13.4 % of pregnant women were beaten on their abdomen during pregnancy in which 86.2 % of the assailants were the biological father of the baby they were carrying, which is slightly higher than WHO finding which was 8 % [3]. This can be explained by inter cultural difference among the two study setting as they are south and west Ethiopia settings. Furthermore, it might be due to the fact that WHO study covered a large section of the population where as this study is in one district. The perpetrator, the biological father (86.2 %) of the baby she was carrying was lower than previous Ethiopian study [3]. This might be due to presence of polygamy in the study area because 37 (13.1 %) of respondents married to partners who had other wife.

One in three, 85 (30 %) of pregnant women had sexual violence during recent pregnancy. This is similar with study conducted in Tanzania [27] which might be due to shared similar socio economic status. It is higher than findings from Namibia [3]. This might be due to sexual autonomy imbalance among the two study areas.

Overall, the prevalence of psychological intimate partner violence during recent pregnancy was 46 (16.3 %) which is lower than study conducted in South Africa which was 49 % [28]. This could be due to difference in the perception of violence in the respective communities [29].

Women who ever lived with their intimate partner’s family were 45 % less likely to experience intimate partner violence during pregnancy when compared with women who had not ever lived with their partner’s family. This might be explained if pregnant women were attached to their husband’s family, their partner’s respect might increase and so lower violence.

Unlike other studies, compared with literates, illiterate partners were 50 % less likely to experience violence by their intimate partner during pregnancy. Pregnant women who reported that dowry payment has positive impact in the rest of their lives were 8.7 times more likely to experience IPVDP than those who reported no positive impact. Pregnant women who didn’t undergo marriage ceremony during their marriage were 4.1 times more likely to experience IPVDP.

Dowry impact on how they are treated by their husband and his family was also a risk factor for IPVDP as dowry is the payment to be made to the groom’s family to marry a daughter, and it takes different forms in different cultures. However, the size of the dowry is a common reason for disputes between the families, with the groom’s family demanding more than the bride’s family can offer, resulting in violence of brides even to death in India and southern Asian countries [30].

## Conclusions

Intimate partner violence during recent pregnancy is observed in nearly half of the pregnant women in the study area. One in three pregnant women experienced both physical and sexual violence. More than one in ten experienced intimate partner psychological/emotional violence. The implication of the study findings regarding the practice of intimate partner violence during recent pregnancy is common in the study site. To that end, increasing community awareness about the consequences and its adverse reproductive health outcomes of the practice could be an important. Moreover, the health extension workers should be engaged in education, screening and referral of IPVDP victims as they are near to the community. In addition the district education office, office of women’s affair should strengthen education on prevention of intimate partner violence in schools. The unique finding from this study is about the association between education and intimate partner violence among pregnant women where it associated positively with less education. To make the associations of demographic variables more clear, we recommend further longitudinal studies to further strengthen the magnitude and associated factors and its effect on pregnant women and the fetus locally.

Moreover, the longitudinal study will help in checking the repeatability of the findings in different areas and with different study design possible to scale it up to incorporate in the government policy.
